# Maternal and Neonatal Outcomes for Egyptian Primigravida Women after Antenatal Dental Health Education Program

**DOI:** 10.1089/whr.2024.0133

**Published:** 2025-07-09

**Authors:** Azza Ibrahim Abd El-Kader, Sabah Abduo Aly Hagrass, Mai Atef Hassan

**Affiliations:** ^1^ Faculty of Nursing, Department of Obstetrics & Gynecological Nursing, Zagazig University, Zagazig City, Egypt.; ^2^ Faculty of Nursing, Department of Community Health Nursing, Zagazig University, Zagazig City, Egypt.; ^3^ Faculty of Dentistry, Periodontology, Oral Diagnosis, and Radiology Department, Tanta University, Tanta City, Egypt.

**Keywords:** Dental health program, Egyptian primigravida women, Maternal outcomes, Neonatal outcomes

## Abstract

**Background:**

Educational programs for pregnant women delivered by dentists and midwives would prevent oral disorders in mothers and also impact the dental health of offspring.

**Aim of the Study:**

The main aim of this study is to evaluate the effect of an antenatal dental health education program on maternal and neonatal outcomes among Egyptian primigravida women.

**Subjects and Methods: Research Design:**

A quasi-experimental research design was carried out. Subjects: 108 pregnant women who met the inclusion criteria. The sample size was two equal groups and divided into two groups (case and control).

**Setting:**

The study was conducted at the prenatal clinic at Zagazig University Hospital.

**Tools of Data Collection:**

The following four tools were used to achieve the study’s purpose: a structured interview questionnaire; a scale for assessing knowledge, practice, and attitude; and an outcome checklist for tracking mother and baby outcomes throughout labor. In addition, prenatal education classes were held to enhance the pregnancy outcomes.

**Results:**

According to the study’s findings, women who participated in the dental education program had statistically significant positive knowledge, practices, and attitudes compared with those in the control group. In the case group, pregnancy outcomes were good and improved after attending the dental health program more than in the control group.

**Conclusion:**

Designing and implementing an educational program on antenatal oral health had a significant effect on improving pregnancy outcomes.

**Recommendation:**

An awareness educational program about dental care associated with pregnancy was necessary to be recommended.

## Introduction

Pregnancy is a period of vulnerability because many physiological changes in the body of pregnant women can affect the oral cavity.^1^ Furthermore, the gestation period presents both physiological and psychological changes that expose the oral cavity to pathologies that can affect the mother’s general health, such as hormonal changes that interfere with the oral cavity and aggravating pathologies, such as periodontal disease, gingivitis, and dental caries. Governments worldwide have adopted international recommendations for dental care during pregnancy since oral health is a key indicator of general health, well-being, and quality.^2^

Gingival inflammation is commonly encountered in the second trimester, and preexisting periodontitis is aggravated during pregnancy.^3^ Additionally, pregnant women who consume cariogenic foods and alter their diet have lower salivary flow, morning sickness, and poor oral hygiene habits, all of which can lead to dental caries and tooth erosion. During pregnancy, dental care was totally neglected, and patients only went to the dentist when they experienced bleeding gums or a toothache. Pregnant women have a higher risk of dental caries, gingivitis and periodontal disease than nonpregnant women.^4^

Worldwide, 50% of pregnant women experience oral pain, and 40% have periodontal disease.^5^ Periodontal disease is the most common dental condition (30%–60%), followed by dental caries and pain (30%–40%).^6^ Periodontal disease during pregnancy is linked to poor birth outcomes such as preterm birth and low birth weight.^7^ Pregnant women with untreated oral infections are more likely to develop sepsis, preeclampsia, and miscarriages. The oral health of pregnant women is also linked to the development of early childhood dental caries in their offspring.^8^

The most frequently reported barriers to dental care utilization during pregnancy were a lack of understanding of the value of oral health care, poor beliefs about the safety of dental treatment during pregnancy, and a lack of perceived need for dental care.^9^ The World Health Organization (WHO) emphasizes the need to integrate oral health with maternity and child health for efficient prevention and management of oral illnesses.^10^ Maintaining oral health care during pregnancy and throughout a woman’s lifespan is crucial for ensuring a healthy pregnancy.^11^

Additionally, it is also important for many health care professionals, including dentists, dental hygienists, physicians’ assistants, public health primary nurses, midwives, obstetricians, and nurse practitioners, to emphasize providing sufficient and timely oral health care to pregnant women, including oral health education programs.^12^ Therefore, establishing relationships between antenatal care and oral health providers in the community facilitates a collaborative approach to women’s oral health needs.^11^

Education programs on dental health and disease prevention are critical for women of reproductive age and for pregnant women. The design of a dental care plan for pregnant women, as well as dental prophylaxis, should be developed and guided by institutional clinical authorities. All health care providers, including primary care, pediatric dentists, and maternity care providers, consider paying more attention to oral health during pregnancy. These collaborations provide excellent antenatal dental teamwork and promote good pregnancy outcomes as well as a future generation of children free from oral illness. Implementation of an educational program that matches with the community, the women’s knowledge, practice, and attitude regarding oral health should be considered.^13^ Therefore, this study aimed to evaluate the effect of an antenatal dental health education program on pregnancy outcomes for Egyptian primigravida women in Zagazig City.

### Aim of the study

The aim of the study was to evaluate the effect of antenatal dental health education program on maternal and neonatal outcomes among primigravida women.

### Research objectives

The research aims of this study were achieved through the following objectives:

1. Determine the effect of prenatal dental health education sessions on women’s knowledge, practices, and attitudes.
2. Evaluate pregnancy outcomes for the case group following participation in an oral health education program.

### Research hypothesis

1. Primigravida women who will receive antenatal dental education sessions will have significantly better knowledge, practice, and attitude toward oral health.
2. Pregnancy outcomes for the primigravida women in the case group will be better than those of primigravida women in the control group after conducting the dental health education program.

## Subjects and Methods

### Research design

A quasi-experimental research approach was used in this study (comparative study).

### Research setting

This study was conducted at the prenatal clinic of the Zagazig University Hospital in Zagazig City, Egypt. This study was conducted between December 2022 and April 2023, from Saturday to Thursday, with the prenatal clinic open every day from 9 a.m. to 2 p.m.

### Study subjects

At the time of data collection, a prospective sample of 108 pregnant women with gestational weeks 24–42 (second and third trimester) was collected. The sample size was divided into two equal groups (n = 54 each) and two groups (case and control).

Based on data from literature,^14^ considering a level of significance of 5%, and study power of 80%, the sample size can be calculated using the following formula:

 
$$\text{n }=\frac{{\left(\text{Zα}/2\;+\text{ Zβ}\right)}^{2}\;\times \;{2(\text{SD})}^{2}\;}{d^{2}}$$

where, SD is the standard deviation obtained from a previous study; Z_α/2_, for 5% this is 1.96; Z_β_, for 80% this is 0.84 and for the expected difference. Therefore, $\text{n }=\frac{{\left(1.96\;+\;0.84\right)}^{2}\;\times \;{2(0.92)}^{2}\;}{{\left(0.5\right)}^{2}}$ =53.1. Based on this formula, the required sample size for each group was 54.

### Inclusion/exclusion criteria

The inclusion criteria for the study group were age 20–40 years, attending antenatal care clinics at Zagazig Hospital at the time of data collection, having a gestation period of 24–42 weeks, pregnant women with dental problems (dental caries and periodontitis), and providing informed consent to participate in the study.

Exclusion criteria included pregnant women who required emergency treatment, including those requiring hospitalization, surgery, or serious infection, and those with a history of mental illness were excluded.

### Tools for data collection

After written consent was obtained, data were gathered through in-person interviews. The following four tools were used and employed by the researchers during the interviews to gather data:

1. A structured *interview questionnaire* that was structured and asked about the following information: women’s age, residence, education level, and occupation. Data on current gestational age and signs and symptoms of gingival and periodontal disease among pregnant women were also collected, as well as obstetric history. In addition, factors that prevent pregnant women from visiting dentists during pregnancy were evaluated.
2. *Clinical oral examination*: Dental caries experience and periodontal status were assessed using WHO’s Oral Health Surveys Basic Methods.^15^ Dental health specialist (M.A.H.) conducted all oral examinations. The examinations were performed in a suitable room at an antenatal clinic under standardized conditions. Dentition status was assessed using plane mouth mirrors and the community periodontal index of treatment needs probes.
3. *A self-administered questionnaire* addressing various aspects of expectant mothers’ knowledge, practices, and attitudes (A, B, and C parts) about oral health and oral hygiene practices. The questionnaire was prepared by the researchers in English and Arabic. The questions were developed after referring to relevant literature to assess the participants’ knowledge, practices, and attitudes. The validity and reliability of the questionnaire were assessed. The final form of the questionnaire was available in English and Arabic and was ready for use by study staff members. This was read to illiterate women. The correlation coefficient test was used to test for correlations between two variables with continuous data. The reliability (internal consistency) of the questionnaire used in this study was calculated.

Part A included questions on oral health knowledge. It includes 16 items; knowledge scores range from 0 to 10; scoring system: scores lower than 50% (lower than 5) are considered *poor*; scores from 50 to <75% (scores from 5 to 7) are considered *fair*; and scores that are 75% or higher (scores from 8 or higher) are considered *good*.

Part B: The following section covers the assessment of the respondents’ daily oral health practices and dental health service utilization during pregnancy, which includes 17 items. Scoring system: Practice scores ranged from 0 to 14; scores lower than 50% (lower than 7) were considered *poor*; scores from 50% to <75% (scores from 7 to 10) were considered *fair*; and scores of 75% or higher (scores from 11 or higher) were considered *good*.

Part C: The last section contained issues related to dental health attitudes, which were set to survey respondents’ attitudes toward oral health, including 13 items. The expectant mothers were asked to indicate their preference as to whether they “strongly agree, agree, are neutral or undecided, disagree, or strongly disagree” in every statement. Scoring system: Attitude scores ranging from 0 to 9 and scores lower than 60% (lower than 5) were considered *unsatisfactory*, and scores of 60% or higher (scores from 5 or higher) were considered *satisfactory*.

4. *Outcome measures* to obtain data on maternal and fetal outcomes at the labor unit, including preterm delivery, presence of preeclampsia, and delivery of delivery of low birthweight newborn (LBW). Preterm birth was defined as a gestational age <37 weeks. LBW was defined as a birth weight <2.5 kg.

### Content validity and reliability

The researchers reviewed local and international literature to obtain more knowledge about the study and designed the study tools. Five experts in obstetrics and gynecological nursing with community health nursing specialist staff and one dentist from the Faculty of Dentistry evaluated the instruments for content validity.

Cronbach’s alpha values for knowledge, practice, and attitude were 0.902, 0.893, and 0.898, respectively.

### Measure methods

The recommended modifications were made, and the final form was ready for use. The researchers visited the study site throughout the study period and checked the registration book to identify the pregnant women who met the inclusion criteria. Each woman was individually met by the researchers, who thoroughly explained the purpose of the study to win their acceptance and their written consent.

After approval of the official permission using proper channels of communication was obtained from the director of the previously mentioned study setting, the researchers attended the antenatal clinics in the study setting 3 days per week from 9:00 am to 2:00 pm for 5 months from the beginning of December 2022 to the end of April 2023. A pretest was conducted by distributing the structured questionnaire after sufficient clarification of the purpose of the study for the pregnant participants in each small homogeneous group.

#### The educational sessions for the studied group

The researchers separated the antenatal education group participants into small groups, each of which consisted of 10 pregnant women. There were three weekly sessions of 45–90 minutes, each using the appropriate teaching methods and aids. Each session started with a summary of what was given in the previous session and the objectives of the new session to ensure that pregnant women recognized the contents of the educational sessions. The first part was to provide a theoretical background on knowledge about oral health care during pregnancy, including one session.

The second part was to provide practical sessions about the practices and attitudes of pregnant women regarding oral health care during pregnancy, such as different methods of oral hygiene, false attitudes associated with oral health, and correction. In addition, the relationship between good oral health care and pregnancy outcomes included two sessions. There was one last session to start and end the antenatal dental health education program on pregnancy outcomes for the revision of all sessions to ensure motivation and reinforcement to enhance the effectiveness of the educational program on the participants’ knowledge, practices, and attitudes regarding oral health during pregnancy. The contents of education classes are presented in Table 1.

**Table 1.** Comparison of the Obstetric History and Barriers of Receiving Dental Care During Pregnancy Between Case and Control Groups

Obstetric history							
	Case		Control		Chi-Square		
	*n*	%	*n*	%	χ^2^	*p*	
Current gestational age (weeks)							
	<25	9	16.7	7	13.0		
	25–34	45	83.3	38	70.4		
35 or more	0	0.0	9	16.6	9.840	0.007^*^	
	Signs and symptoms of gingival and periodontal disease among pregnant females						
	Swelling gum	25	46.3	30	55.5		
	Bleeding gum	15	27.7	10	18.5		
	Loose teeth	6	11.1	5	9.3		
	Pus formation	3	5.6	5	9.3		
No problems	5	9.3	4	7.4	2.157	0.707	
Factors that hinder the expecting women from visiting the dentist during pregnancy							
Lack of time	15	24.2	26	23.2	4.757	0.029^*^	
Cost of dental services	13	20.9	23	20.5	4.167	0.041^*^	
Fear and anxiety	22	35.5	34	30.4	5.571	0.018^*^	
Transportation problem	8	12.9	17	15.2	4.216	0.040^*^	
Habit	4	6.5	12	10.7	4.696	0.030^*^	

N.B.: The participants chose more than one answers regarding factors that hinder the women from visiting the dentist.

Data are presented as *n* (%). *p*-Value based on Monte Carlo exact probability.

^*^ *p* < 0.05 (significant).

χ^2^; Chi-square test.

The health education dental care program sessions were implemented through various teaching methods, such as short lectures, interactive group discussions, brainstorming, demonstrations, re-demonstrations, and role-play. The researchers prepared illustrative, comprehensive instructional media, such as handbooks in simple Arabic language and posters; PowerPoint presentations were used to conduct antenatal dental care education classes. Data on knowledge, practices, and attitudes were collected at the conclusion of the third class.

PowerPoint presentations, posters, and hand booklets were given to the participants to be used later and to help in awarding other relative personnel. The contents included well-formed information about oral health care during pregnancy, including the definition, signs and symptoms, causes, risk factors, treatment, adverse effects, preventive measures for periodontitis, and the importance of dental care during pregnancy.

#### Evaluation phase

The final post-intervention educational test was performed immediately after the end of the educational session, and after 4 months of administering the educational session, follow-up was performed using the same pretest tool.

The control group received routine antenatal care and was exposed to all conditions of the antenatal education group except for the antenatal education sessions. Information was gathered from the control group at the same time as the antenatal appointments.

### Pilot study

A pilot study was carried out on 10% of the overall sample size to check the tools for clarification, applicability, and viability, and then the required adjustments were made.

### Administration and ethical considerations

Oral and written consent was obtained from the women who wanted to participate in this study. All procedures, including the human members, were in accordance with the ethical principles of the institutional and/or national research committee, as well as the 1964 Helsinki Declaration and its later corrections, or tantamount moral measures. This study was approved by the ethics committee. The anonymity, confidentiality, and privacy of the participants were assured, and voluntary participation and the right to refuse to participate in the study were assured at any time during the study period. This clinical trial was affirmed by the Ethical Committee of the Faculty of Nursing at Zagazig University, with the ethical number Zu.Nur.REC#:0060.

### Statistical analysis

All statistical analyses were performed using SPSS for Windows version 20.0 (SPSS, Chicago, IL, USA). Continuous data were normally distributed and expressed as the mean ± SD. Categorical data are expressed as numbers and percentages. A one-way analysis of variance test was used to compare more than two variables with continuous data. The chi-square test (or Fisher’s exact test when applicable) was used to compare variables with categorical data. Statistical significance was set at *p* < 0.05.

## Results

Table 2 shows the distribution of the women investigated based on their demographic variables. The mean ± SD values for age were 24.7 ± 3.7 and 25.6 ± 4.0 for case and control, respectively. Furthermore, no discernible difference in university education levels existed between 55.5% of the women in the case group and 42.5% of the women in the control group. According to the same table, nearly two-thirds (59.3%) of the women in the case group were housewives, with 53.7% coming from rural regions, compared with 70.4% of the housewives and 61.1% living in rural areas in the control group.

**Table 2.** Comparison of Pre-Intervention Knowledge, Practice, and Attitude Between Case and Control Groups

Variables	Case		Control		Chi-Square	
	*n*	%	*n*	%	χ^2^	*p*
Knowledge level						
Poor knowledge	37	68.5	38	70.4		
Fair knowledge	13	24.1	12	22.2		
Good knowledge	4	7.4	4	7.4	0.053	0.974
Practice level						
Poor practice	39	72.2	42	77.8		
Fair practice	11	20.4	7	13.0		
Good practice	4	7.4	5	9.2	1.111	0.574
Attitude level						
Negative attitude	44	81.5	47	87.0		
Positive attitude	10	18.5	7	13.0	0.628	0.428

Data are presented as *n* (%). *p*-Value based on Monte Carlo exact probability.

Table 3 shows that there was a statistically significant difference in the obstetric history of the two groups. In the experimental group, 70.4% of women were between 25 and 34 weeks of pregnancy, compared with 83.3% in the control group. In the experimental group, 46.3% of women reported swollen gums, compared with 55.6% of women in the control group. Other signs and symptoms of gingival and periodontal disease found in pregnant females were bleeding per gum (27.7%) and loose teeth (11.1%) in the case group compared with bleeding per gum (18.5%) and loose teeth (9.3%) in the control group. Furthermore, in this table, the primary variables that prevent the investigated sample from visiting the dentist during pregnancy were fear and anxiety, a lack of time, the high cost of dental services, and a transportation difficulty, and the differences were statistically significant between two groups (*p* < 0.05).

**Table 3.** Comparison of the Knowledge, Practice, and Attitude Between Case and Control Groups at Post-Intervention

	Case		Control		Chi-Square	
	*n*	%	*n*	%	χ^2^	*p*
Knowledge level						
Poor knowledge	6	11.1	32	59.2		
Fair knowledge	10	18.5	15	27.8		
Good knowledge	38	70.4	7	13.0	40.145	<0.001^*^
Practice level						
Poor practice	8	14.8	35	64.8		
Fair practice	12	22.2	10	18.5		
Good practice	34	63.0	9	16.7	31.670	<0.001^*^
Attitude level						
Negative attitude	13	24.1	43	79.6		
Positive attitude	41	75.9	11	20.4	33.379	<0.001^*^

Data are presented as *n* (%). *p*-Value based on Monte Carlo exact probability.

^*^ *p* < 0.05 (significant).

Table 4 reveals that low knowledge (68.5%), fair knowledge (24.1%), and excellent knowledge (7.4%) were reported prior to the delivery of the oral health program in the case group. The control group had poor knowledge (70.4%), reasonable understanding (22.2%), and excellent knowledge (7.4%). Variations in knowledge, practice, and attitude levels before the education program administration were not statistically significant between the control and case groups.

**Table 4.** Outline for 3-Week Antenatal Dental Health Education Sessions

Weeks	Contents	Objectives
First class (1st week)	Knowledge about oral health care during pregnancy	1. Introduce everyone to one another and foster rapport.
		2. Describe the goals of antenatal dental health education sessions.
		3. Introduction to pretest questionnaires to assess knowledge level.
		4. Illustrate the different methods of oral hygiene as brushing teeth, using dental floss, mouth fresheners, and using mouth wash.
		5. Describe the safe period during pregnancy for management of oral disease.
		6. Discuss the effect of radiation on pregnancy.
		7. Distribute post-test.
Second class (2nd week)	Practice and attitude regarding oral health care during pregnancy	1. Review content from class one.
		2. Introduction to pretest questionnaires to assess practice and attitude level.
		3. Perform the different methods of oral hygiene as brushing teeth, using dental floss, mouth fresheners, and using mouth wash.
		4. Identify the false attitude associated with oral health and correct it.
		5. Perform post-test to determine the effectiveness of education program.
Third class (3rd week)	Relation between good oral health care and maternal and neonatal outcomes	1. Review the main items of the previous class.
		2. Distribute pretest.
		3. Determine the factors that hinder the pregnant women from visiting dentist.
		4. Explain the adverse maternal and neonatal outcomes associated with bad oral hygiene.
		5. Illustrate the reasons for adverse maternal and neonatal outcomes.
		6. Administer post-test, finally.

Designed by researchers of the main article after revision of national and international related articles: this table is designed according to education classes that are given to pregnant women.

Table 5 reveals that after receiving an education program, the investigated women had statistically greater knowledge, excellent practice, and a positive attitude than the control group. Following the delivery of the dental health program, the case group had poor (11.1%), fair (18.5%), and good (70.4%) knowledge. Regular care revealed that the control group had poor knowledge (59.2%), fair knowledge (27.8%), and excellent knowledge (13.0%).

**Table 5.** Comparison of the Demographic Characteristics Between Case and Control Groups

Variables	Case		Control		Chi-square	
	*n*	%	*n*	%	χ^2^	*p*
Age (years)						
20–25	36	66.6	30	55.6		
26–30	13	24.1	16	29.6		
31–35	5	9.3	8	14.8	1.548	0.461
Mean ± SD	24.7 ± 3.7		25.6 ± 4.0		1.187	0.238
Residence						
Rural	29	53.7	33	61.1		
Urban	25	46.3	21	38.9	0.606	0.436
Educational status						
Illiterate	4	7.4	7	13.0		
Primary	5	9.3	7	13.0		
Secondary	15	27.8	17	31.5		
University	30	55.5	23	42.5	2.201	0.532
Occupation						
Housewife	32	59.3	38	70.4		
Employee	22	40.7	16	29.6	1.462	0.227

Data are presented as *n* (%). *p*-Value based on Monte Carlo exact probability.

SD, standard deviation.

**FIG. 1.**
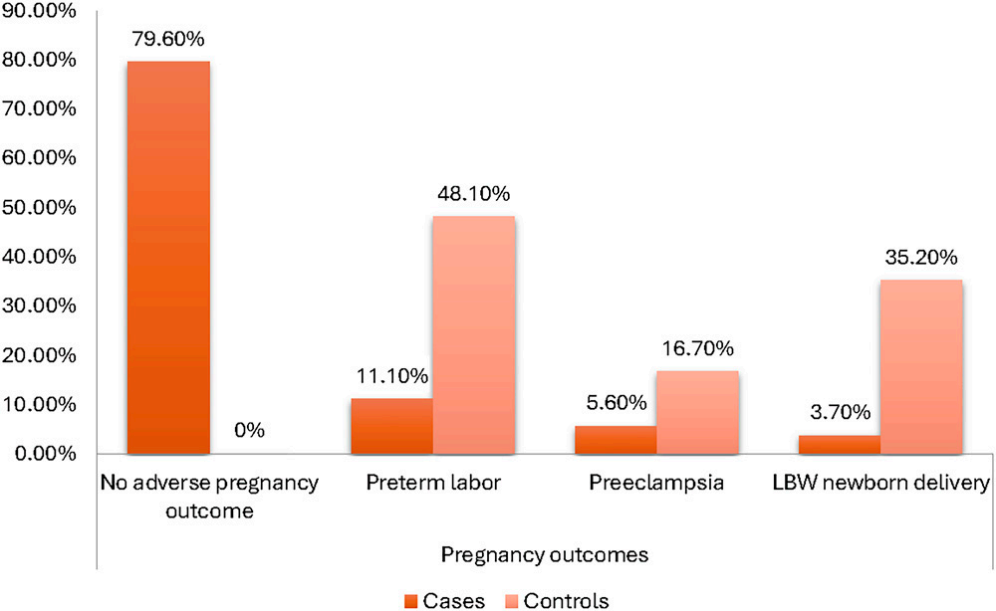
Pregnancy outcomes between case and control groups.

Figure 1 depicts the pregnancy outcomes following the provision of an education program for the case group and usual treatment for the control group. The outcomes were as follows: no adverse outcomes, preterm labor, preeclampsia, and LBW birth in the case group (79.6%, 11.1%, 5.6%, and 3.7, respectively). In the control group, the pregnancy outcomes included no adverse outcomes (0.0%), preterm labor (48.1%), preeclampsia (16.7%), and LBW (35.2%). The differences in pregnancy outcomes between the two groups analyzed were statistically significant (*p* < 0.001).

## Discussion

Research on enhancing women’s awareness of oral health care in Africa is uncommon because of the lack of priority. Various studies have revealed that maternal oral health is poor and that oral hygiene in pregnant women is hampered by various additional obstacles to achieving optimal oral health. Thus, educating pregnant women about oral health can be an effective approach for delivering dental health education to the entire population, beginning at the individual level and progressing to family and community levels, as mentioned by Chawla et al.^16^

In this study, the mean ± SD for age in the case and control groups was between 20 and 40 years. Furthermore, there was no discernible difference between the university education levels of the women in the case group and the women in the control group. This is congruent with research conducted by Abd El-Kader.^17^ This explains the reproductive age and why people were interested in participating in this study.

The current study aimed to improve pregnant women’s knowledge, practice, and attitude toward dental health care and pregnancy outcomes, which were significantly improved because there were statistically significant improvements in women’s knowledge of dental care and improved pregnancy outcomes compared with primiparous women who received routine antenatal care. Following the intervention session, most of the participating women corrected their understanding. This might be attributed to the use of simple and clear language in educational sessions, the suitable teaching techniques, and multimedia aids.

This was consistent with a study conducted in India that emphasized how periodontitis affects maternal health and pregnancy outcomes, stating that there is a need for more forceful awareness sessions to help spread the message of periodontitis’s adverse effects on pregnancy and the importance of oral health hygiene to establish proper healthy habits and prevent oral disease.^18^ Similarly, the necessity of educational programs was highlighted during an investigation of pregnant women’s oral health hygiene in Brazil and its relationship with lifestyle health practices.^19^

Similarly, this study was in accordance with the studies done in Sudan (Khаrtoum), Fayoum (Egypt), Assiut (Egypt), and Benhа (Egypt)^20–23^ on pregnant women. These studies concluded that pregnant women’s oral health education should be improved, and oral health preventative programs should be implemented to improve pregnancy outcomes, such as premature labor, which is associated with poor oral health. Therefore, maternal periodontal diseases may constitute a non-genital source of entry into the circulatory system for bacteria, with the potential to impair fetal–maternal health.

Preterm delivery and preeclampsia have been linked to maternal inflammation. Periodontal disorders have been linked to premature births and preeclampsia in several studies. The principal causative agent of dental caries is *Streptococcus mutans*. Consequently, untreated dental caries may lead to further inflammatory issues, which may have an impact on pregnancy outcomes. Similarly, we hypothesized that dental caries would be linked to preterm delivery and preeclampsia *via* an infectious mechanism; however, we discovered no such link, which contradicts previous research by Mousa et al.^3^

In the current study, the primary variables preventing the examined sample from visiting the dentist during pregnancy were fear and anxiety, lack of time, high cost of dental services, and transportation issues; the differences were statistically significant between the two groups (*p* < 0.05). These findings are similar to those published by Khalaf et al.,^23^ who indicated that lack of information or sufficient access to health care institutions that disseminate optimal oral health and hygiene during pregnancy might be the likely cause. This study emphasizes the need to educate expecting mothers because they are responsible for their own health as well as the health of their children.

Finally, pregnant women should be taught about the link between periodontal disease and adverse pregnancy outcomes and should be encouraged to undergo frequent dental checkups. Furthermore, doctors and health centers should undertake nutritional education programs for pregnant women, with a focus on women from rural regions. In addition, if a woman is contemplating pregnancy or is pregnant, she should undergo frequent check-ups and treatments for tooth caries without the need for dental treatment. Future plans should also incorporate oral health literacy into children’s school curricula, allowing them to become literate adults in the long term (EL Sayed and Said).^24^

## Conclusions

The majority of the women who participated in the study had little awareness of the relationship between poor oral health and pregnancy outcomes. The design and implementation of an educational program on the subject resulted in a substantial increase in the participants’ level of knowledge, practice, and attitude toward it. The changes in pregnancy outcomes between the two groups were statistically significant, with improved pregnancy outcomes compared within the study group.

### Recommendations

Based on the study findings, the study is recommending the following:

Conducting an awareness educational program about dental care and periodontitis that is associated with pregnancy outcomes was necessary to be implemented as a component of the services that are provided to the pregnant women in the antenatal care clinics that should mainly involve women from all residence areas, particularly in Egypt’s rural communities. Reinforce routine oral health maintenance and dental visits twice a year, as research shows poor dental health could affect the general oral health of a developing baby. Ensure health coverage for dental services during pregnancy by applying health education campaigns during prenatal care to increase awareness of oral health among pregnant women and improve oral health practices and attitudes, as well as those referrals that can be made.

Nursing programs and curricula need to change to prepare and train nursing graduates with core competencies of oral health access to care issues, as they are the main oral health educators and care providers at the different primary health care settings of the communities. Advocate for broader oral health coverage of women before, during, and after pregnancy through oral health promotion programs for pregnant women and use available and appropriate ways and creative, consistent, and comprehensive communication strategies. Further research may be conducted to study the same problem using large samples of the women with long-term follow-up.

## Abbreviations Used

LBW: Low birth weight
n: Sample size
SD: Standard deviation
WHO: World Health Organization
